# Anti-neuroinflammatory potential of *Tylophora indica* (Burm. f) Merrill and development of an efficient *in vitro* propagation system for its clinical use

**DOI:** 10.1371/journal.pone.0230142

**Published:** 2020-03-25

**Authors:** Vasudha Gupta, Rupam Guleri, Muskan Gupta, Navdeep Kaur, Kuldeep Kaur, Paramdeep Kumar, Manju Anand, Gurcharan Kaur, Pratap Kumar Pati

**Affiliations:** 1 Department of Biotechnology, Guru Nanak Dev University, Amritsar, Punjab, India; 2 Amity Institute of Biotechnology, Amity University, Haryana, India; National University of Kaohsiung, TAIWAN

## Abstract

Neuroinflammation is a major risk factor associated with the pathogenesis of neurodegenerative diseases. Conventional non-steroidal anti-inflammatory drugs are prescribed but their long term use is associated with adverse effects. Thus, herbal based medicines are attracting major attraction worldwide as potential therapeutic candidates. *Tylophora indica* (Burm. f) Merrill is a valuable medicinal plant well known in Ayurvedic practices for its immunomodulatory, anti-oxidant, anti-asthmatic and antirheumatic activities. The present study aimed to elucidate the anti-neuroinflammatory potential of water and hydroalcoholic leaf extracts of micropropagated plants of *T*. *indica* using BV-2 microglia activated with lipopolysaccharide as an *in vitro* model system and development of an efficient reproducible protocol for its *in vitro* cloning. Non cytotoxic doses of the water and hydroalcoholic extracts (0.2μg/ml and 20μg/ml, respectively) were selected using MTT assay. α-Tubulin, Iba-1 and inflammatory cascade proteins like NFκB, AP1 expression was studied using immunostaining to ascertain the anti-neuroinflammatory potential of these extracts. Further, anti-migratory activity was also analyzed by Wound Scratch Assay. Both extracts effectively attenuated lipopolysaccharide induced microglial activation, migration and the production of nitrite via regulation of the expression of NFκB and AP1 as the possible underlying target molecules. An efficient and reproducible protocol for *in vitro* cloning of *T*. *indica* through multiple shoot proliferation from nodal segments was established on both solid and liquid Murashige and Skoog’s (MS) media supplemented with 15μM and 10μM of Benzyl Amino Purine respectively. Regenerated shoots were rooted on both solid and liquid MS media supplemented with Indole-3-butyric acid (5–15μM) and the rooted plantlets were successfully acclimatized and transferred to open field conditions showing 90% survivability. The present study suggests that *T*. *indica* may prove to be a potential anti-neuroinflammatory agent and may be further explored as a potential therapeutic candidate for the management of neurodegenerative diseases. Further, the current study will expedite the conservation of *T*. *indica* ensuring ample supply of this threatened medicinal plant to fulfill its increasing demand in herbal industry.

## Introduction

*Tylophora indica* -an important medicinal plant belonging to family Asclepidaceae is a perennial climber which is considered as one of the best indigenous substitute for ipecacuahna [1]. It is commonly known as “Antmool” and is traditionally used as a folk remedy in the treatment of asthma, allergy, jaundice, rheumatism and other respiratory problems [2, 3]. It has also been reported to possess anti-cancer, immunomodulatory, hepatoprotective, antidiabetic and antioxidant activities [4, 5, 6, 7, 8]. Medicinal potency possessed by this herb is attributed to both alkaloid and non-alkaloid constituents which have various therapeutic effects. The roots and leaves of *T*. *indica* contain various active alkaloids such as tylophorine, tylophorinine, anticancerous tylophrinidine and a number of other non alkaloid components such as septidine, isotylocereberine, sterols, and flavonoids [9, 10, 11].

Nowadays, prevalence of neurodegenerative diseases (NDDs) like Alzheimer, Parkinson, multiple sclerosis ‘etc’. are increasing at an alarming rate, therefore, imposing substantial burden on healthcare systems worldwide. Numerous experimental and clinical studies have provided evidence that neuroinflammation is the unifying critical factor responsible for onset and progression of these diseases among their distinct pathological features [12, 13]. Brain immune cells, especially microglial cells are the key cellular players whose uncontrolled over activation leads to generation of self-perpetuating inflammatory responses, ultimately resulting in neurodegeneration and cognitive deficits associated with these NDDs. Normally the microglial cells play important role in immune defense and help in maintaining the CNS homeostasis. In response to external stimuli, they undergo phenotypic switch from resting ramified to activated amoeboid morphology and secretes various inflammatory mediators like cytokines, reactive oxygen and nitrogen species, damaged protein products ‘etc’ [14, 15]. These morphological, biochemical and molecular alterations are detrimental for neurons. Further these mediators amplify the inflammatory responses by initiating the self propelling cycle of microglial activation, production of inflammatory products and neuronal damage. Due to complex multi-factorial mechanisms of these progressive NDDs pathogenesis, their therapeutic intervention is a major challenge. Conventionally, the non steroidal anti-inflammatory drugs are prescribed but they alleviate only the symptoms of the disease without treating the disease [16]. Additionally, their long term usage is also associated with several side effects like gastrointestinal problems, dizziness, headache ‘etc’ [17]. Nowadays natural products are emerging as the promising therapeutics because of their pleiotropic nature, economic sustainability and least side effects [18]. *T*. *indica* alkaloids mixture in the form of crude extract from its leaves was found to inhibit the cellular immune response in rats against sheep red blood cells when administered with these cells before and after immunization [19]. Its alkaloid tylophorine was also reported to inhibit the lipopolysaccharide/IFNγ induced production of nitrite in RAW264.7 macrophages [5, 20]. Various studies have reported and evaluated different therapeutic roles of *T*. *indica* in different model systems but no literature reports are available on the anti-inflammatory potential of *T*. *indica* leaves against lipopolysaccharide (LPS)-induced neuroinflammation. In view of this lacuna, the current study was planned to investigate the anti-neuroinflammatory activity of hydroalcoholic and water extract from leaves of this plant against the LPS induced inflammatory responses using murine BV-2 microglial cells as *in vitro* model system.

According to world health organization (WHO), almost 80% of the population in developing nations is dependent upon the use of herbal products for their primary health care needs and their demand is increasing further [21]. Moreover, it is expected that 40% of the compounds used in pharmaceuticals are directly extracted from the plants as their chemical synthesis is economically not feasible [22]. Because of these issues, the market demand of herbal plants has increased worldwide resulting in overexploitation and decline in their natural habitat [23]. Due to the indiscriminate use of *T*. *indica* owing to its huge medicinal potential, this plant has also been listed as one of the threatened plant species in India [24]. Traditionally, *T*. *indica* is propagated through seeds but they have low seed viability and germination and being cross pollinated, the seed progenies are highly heterozygous. Likewise, the plant is poorly amenable to vegetative propagation, thus, limiting multiplication of desired cultivars. [25]. Thus, to meet the increasing demand of *T*. *indica*, it is mandatory to develop alternative methods having high multiplication rates which will substantially reduce the dependence on the wild populations. In this context, *In vitro* propagation using advanced biotechnological tools can be highly beneficial to meet the rising demand of this herb in future [26]. Earlier, the micropropagation of *T*. *indica* has been reported using different explants through axillary shoot proliferation nodal explants and *de novo* adventitious shoot formation either directly from the explants or indirectly through the callus [25, 27, 28, 29, 30, 31]. However, the rate of shoot multiplication using axillary buds was lower as compared with the callus mediated shoot organogenesis. Moreover, there is hardly any information on the micropropagation of *T*. *indica* using liquid culture. Hence, it is imperative to develop efficient micropropagation methods that will result in higher rate of shoot multiplication of *T*. *indica* to fulfill its rising demand. Therefore, the present study was further extended to develop an efficient *in vitro* mass propagation of *T*. *indica* through forced axillary branching using both solid and liquid culture systems.

## Materials and methods

### Anti-neuroinflammatory potential of *T*. *indica*

#### Preparation of *T*. *indica* leaf extract

Leaves were collected from 4 months old *in vitro* raised plants of *T*. *indica* kept in the green house, shade dried and powdered. The aqueous (TWE) and hydroalcoholic (THyE) leaf extracts were prepared by suspending 10g of leaf powder in 100 ml of distilled water and hydroethanol in 1:1 ratio, respectively. The leaf suspensions were incubated overnight at 45°C in shaker incubator with gentle shaking. Following incubation, the suspensions were filtered with Whatmann-1 filter paper under sterile conditions to obtain respective extracts. Aqueous extract obtained was treated as 100% and diluted in Dulbecco’s modified eagle’s medium (DMEM) with 10% fetal bovine serum (FBS) as per experimental requirements. Hydroalcoholic extract obtained was further dried at 45°C and reconstituted in DMSO at the concentration of 50mg/mL and used as per the requirements.

#### BV-2 cell culture and treatments

BV-2 cell line was procured from National Brain Research Centre (NBRC), Manesar, Haryana, India. BV-2 cells were the immortalized murine-cultured microglial cells obtained after transfection with a v-raf/v-myc recombinant virus. The cells were maintained in DMEM supplemented with 10% FBS (Biological Industries) and 1X PSN (GIBCO) at 37°C in 5% CO_2_ humidified environment. When BV-2 microglial cultures reached 80–90% confluency, they were subcultured by 0.01% tyrpsinization and seeded on poly-L-lysine (PLL) coated 12 or 24 well plates as per experimental requirements. The cultures were pretreated with *T*. *indica* leaf extracts i.e. THyE and TWE for 2h before challenging with LPS at the concentration of 100ng/mL [32]. Final treatment was given for 36h before harvesting the cells for different assays.

#### MTT assay

*T*. *indica* extracts were tested for their cytotoxicity using 3-(4, 5)-dimethylthiazol-2-yl)-2,5-diphenyltetrazoliumbromide (MTT) assay. The cell viability was quantified by the conversion of yellow MTT by mitochondrial dehydrogenases of live cells to purple MTT formazan crystals. Further, the crystals formed were solubilized by DMSO and optical absorbance was measured at 594nm wavelength. Briefly for MTT test, the BV-2 cells were seeded in 96 well plate at seeding density of 15,000 cells/ml and treated with different concentrations of THyE and TWE for 48h. After treatment, culture media was replaced by MTT solution (5mg/10mL in serumless media) which was removed after 2h and DMSO was added to dissolve the formed formazan crystals, quantified and analyzed for cell viability.

#### Immunocytochemistry

Control and treated BV-2 microglial cells were washed thrice with 1X PBS followed by fixation with acetone and methanol in 1:1 ratio. Fixed cells were then permeabilized with 0.3% PBST (Triton X-100 in 1X PBS). After permeabilization, the cells were blocked with 2% BSA in 1X PBS and incubated with primary antibodies in 1:250 dilution for 24h in humid chamber (mouse monoclonal anti-Iba-1, anti-NFκB and anti-α-tubulin and rabbit polyclonal anti-AP1 (Sigma-Aldrich, St. Louis, MO, USA)). After 2 or 3 washings with 0.1% PBST, cells were incubated with respective secondary antibodies (i.e AlexaFluor labeled anti-mouse IgG 488 (for α-tubulin, Iba-1 and NFκB) and anti-rabbit IgG 488 (for AP1) for 2 h at room temperature. Cells were stained with an AT-rich region-specific fluorescent nucleus stain, i.e., 4′, 6-diamidino- 2-phenylindole (DAPI) in 1:5000 dilution in 1X PBS for 10 min to study the nuclear morphology. Then the cells were washed twice or thrice with 0.1% PBST and mounted on the slide using anti-fading reagent (Fluoromount, Sigma-Aldrich, St. Louis, MO, USA). Images were captured using Nikon A1R Confocal Laser microscope and analyzed using NIS elements AR analysis software version 4.11.00. Differential interference contrast (DIC) channel images were used to analyze cell morphology. It was also analyzed by Phase contrast images captured using Phase Contrast Inverted Microscope (Nikon TE2000). Experiments were carried out in triplicates.

#### Wound scratch assay

Wound scratch assay was performed to test the anti-migratory potential of *T*. *indica* extracts on LPS activated BV-2 microglial cells using protocol as described in Gupta and Kaur, (2016). For this assay, cells were grown to confluent monolayer, wounded and pretreated with *T*. *indica* extracts followed by activation with LPS. Migration of cells in the scratched area was analyzed as percentage gap size by measuring the distance covered by the invading cells. Experiments were performed in triplicates.

#### Nitrite determination

Production of nitrite was assayed using Griess reagent nitrite determination kit (Molecular Probes, Invitrogen) as per the manufacturer’s protocol. Briefly, the cells were seeded and treated as per treatment regimen. After treatment, culture conditioned medium was collected, centrifuged and mixed with the equal quantity of Griess reagent. The colorimetric reaction was measured at 548nm wavelength and the nitrite concentrations were calculated using the sodium nitrite standard curve.

### Micropropagtion of *T*. *indica*

#### Plant material and explant sterilization

The young and healthy shoots of *T*. *indica* were collected from the healthy and mature plants growing in the Department of Biotechnology, Guru Nanak Dev University, Amritsar, Punjab, India. They were washed under running tap water for 30min and then washed with liquid detergent (1% v/v) for another 10-15min followed by their repeated washing with water to remove the detergent. The explants were thereafter treated 0.4% sodium hypochlorite for 15min. After washing, they were treated with 0.1% of mercuric chloride for 5min and finally rinsed 4–5 times with sterile distilled water. Thereafter, the shoot segments were cut into single node segments (measuring 5-7mm) each holding one dormant lateral bud for inoculation.

#### Culture media and growth conditions

The nodal segments were then planted on Murashige and Skoog’s medium [33] referred to as MS medium augmented with different cytokinins, 3% sucrose and 0.8% agar. The pH of the medium was adjusted to 5.8 before the addition of solidifying agent and the medium was autoclaved at 15psi (121°C) for 20 min. Regenerated shoots were rooted on a separate root inducing medium comprising of IBA supplemented MS medium. All the cultures were incubated in an air conditioned culture room at 25±2°C with a photoperiod of 16h per day with an illumination of 50μmol/m^2^/s^1^ at the level of cultures. Twenty replicates were set for each treatment and each experiment was repeated twice.

#### *In vitro* shoot proliferation on solid and liquid media

For the optimization of shoot proliferation on solid medium, the nodal explants were inoculated on the MS medium fortified with different concentrations of cytokinins like BAP (1.0–15.0μM) and kinetin or KN (1.0–15.0μM). For raising liquid cultures, the liquid MS medium containing sucrose with different concentrations of BAP (0–20μM) was selected for shoot proliferation in conical flasks. The different growth parameters such as shoot length, biomass and shoot numbers were recorded after 4 weeks.

#### Rooting of shoots and acclimatization of *in vitro* raised plantlets

For root induction, the microshoots (4-5cm in height) were inoculated upright in both agar gelled and liquid MS medium containing different auxins including IBA, Indole-3-Acetic Acid or IAA and Naphthalene Acetic Acid or NAA (1–15μM).The basal MS medium without any growth regulators was used as control. For acclimatization, the rooted plantlets were carefully rescued from the culture vessels and transferred to pots containing sand and soil mixture and vermicompost in the ratio of 1:1. Initially, the plantlets were covered with perforated polythene bags and were kept in the growth room under continuous light. After 35 days, the polythene bags were removed and the hardened plants were transferred to earthen pots containing only garden soil and transferred to green house before their final transfer to the full sunlight outdoor.

#### Genetic fidelity analysis using RAPD

The genetic fidelity of micropropagated plants was accessed using RAPD approach. DNA was isolated from the leaf explants of mother plant and *in vitro* raised hardened plants using cetyl trimethyl ammonium bromide (CTAB) method. The quality of the DNA was checked on 1% agarose gel and was quantified using Nanodrop™ spectrophotometer. A total of 14 RAPD primers were used for the screening of plants on the basis of number of scorable and reproducible bands obtained on DNA amplification (Table 1). Among 14 primers, OPA6, OPB10 and OPC8 were used for subsequent analysis as they produced maximum number of scorable and reproducible band patterns.

**Table 1:** Number of amplification products generated with the use of RAPD primers for the analysis of clonal fidelity of *T*. *indica* microshoots.

S.No.	Primer	No. of bands
1.	OPA-05	11
2.	OPA-06	11
3.	OPA-08	3
4.	OPA-14	4
5.	OPB-03	8
6.	OPB-10	12
7.	OPB-13	7
8.	OPC-08	11
9.	OPC-10	8
10.	OPC-15	8
11.	OPD-12	8
12.	OPD-20	4
13.	OPG-03	10
14.	OPH-02	9

#### Statistical analysis

Data is presented as mean ± standard error of the values obtained from at least three independent experiments. The Sigma Stat for Windows (version 3.5) was used to analyze the results by one-way ANOVA (Holm-Sidak post hoc and Tukey’s method) in order to determine the level of significance of the mean values. Values of p ≤ 0.05 were considered as statistically significant.

## Results

### Standardization of effective dose of THyE and TWE

**Fig 1:**
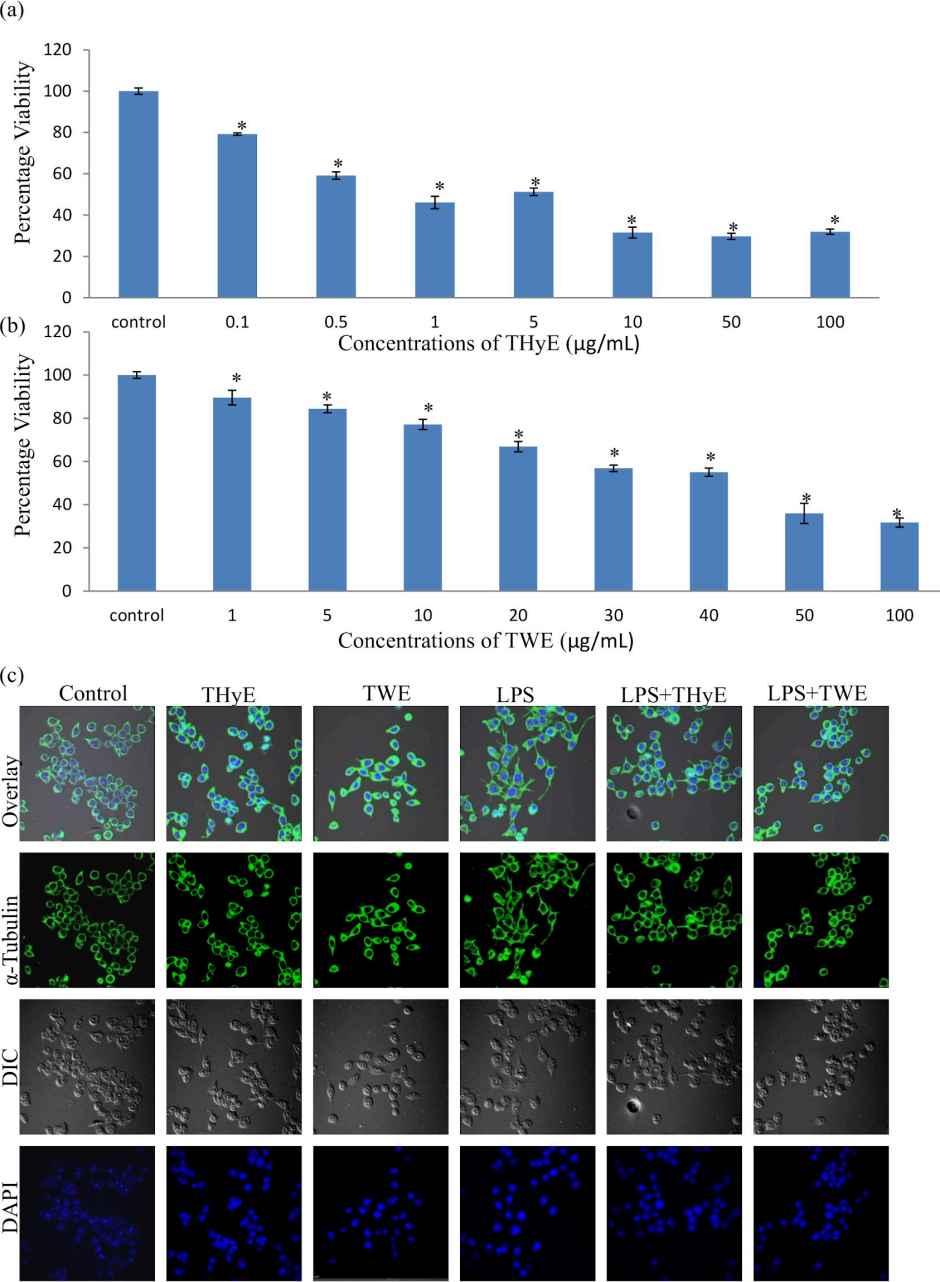
THyE and TWE pretreatment inhibit LPS induced morphological changes in BV-2 microglial cells. (a, b) Histograms depicting the cytotoxicity assay of the BV-2 microglial cells with (a) THyE and (b) TWE after 48 h of treatment. (c) Confocal images of α-tubulin immunostaining of the BV-2 microglial cells pretreated with THyE (0.2 μg/mL) and TWE (20 μg/mL) with or without activation with LPS presenting morphological changes induced by LPS treatment (Scale bar 50μm). Images were captured using A1R Nikon Confocal laser scanning microscope at 60X objective magnification. *p ≤ 0.05 represents statistically significant difference between control and treated groups in MTT assay.

To determine the effective concentration of THyE and TWE, BV-2 microglial cells were treated with different concentrations of these extracts in the range of 0.1–100μg/mL and 1–100μg/mL, respectively for 48h. The cell viability was quantified by the conversion of yellow MTT into formazan crystals. The IC_50_ value was observed at 0.5μg/mL and 15μg/mL for THyE and TWE, respectively (Fig 1A and 1B). On the basis of this preliminary data, maximum nontoxic doses, i.e., 0.2μg/mL and 20μg/mL were used for THyE and TWE, respectively, for further experiments.

### *T*. *indica* extracts and microglial morphology

In response to any danger or toxic stimuli, microglial cells undergo morphological changes from normal ramified to activated amoeboid morphology. Changes in the morphology were studied by cytoskeletal protein α-tubulin immuostaining. It was observed that LPS treated BV-2 microglial cells showed amoeboid morphology with enlarged cellular soma along with filopodia like cytoplasmic processes. Pretreatment of activated BV-2 microglial cells with THyE and TWE was seen to prevent LPS induced morphological changes and cells showed normal morphology like control cells. THyE and TWE alone treatment did not induce any morphological changes in BV-2 microglial cells. Differential interference contrast images clearly denoted these phenotypic changes in LPS activated BV-2 cells (Fig 1C).

### T. *indica* extracts and microglial migration

**Fig 2:**
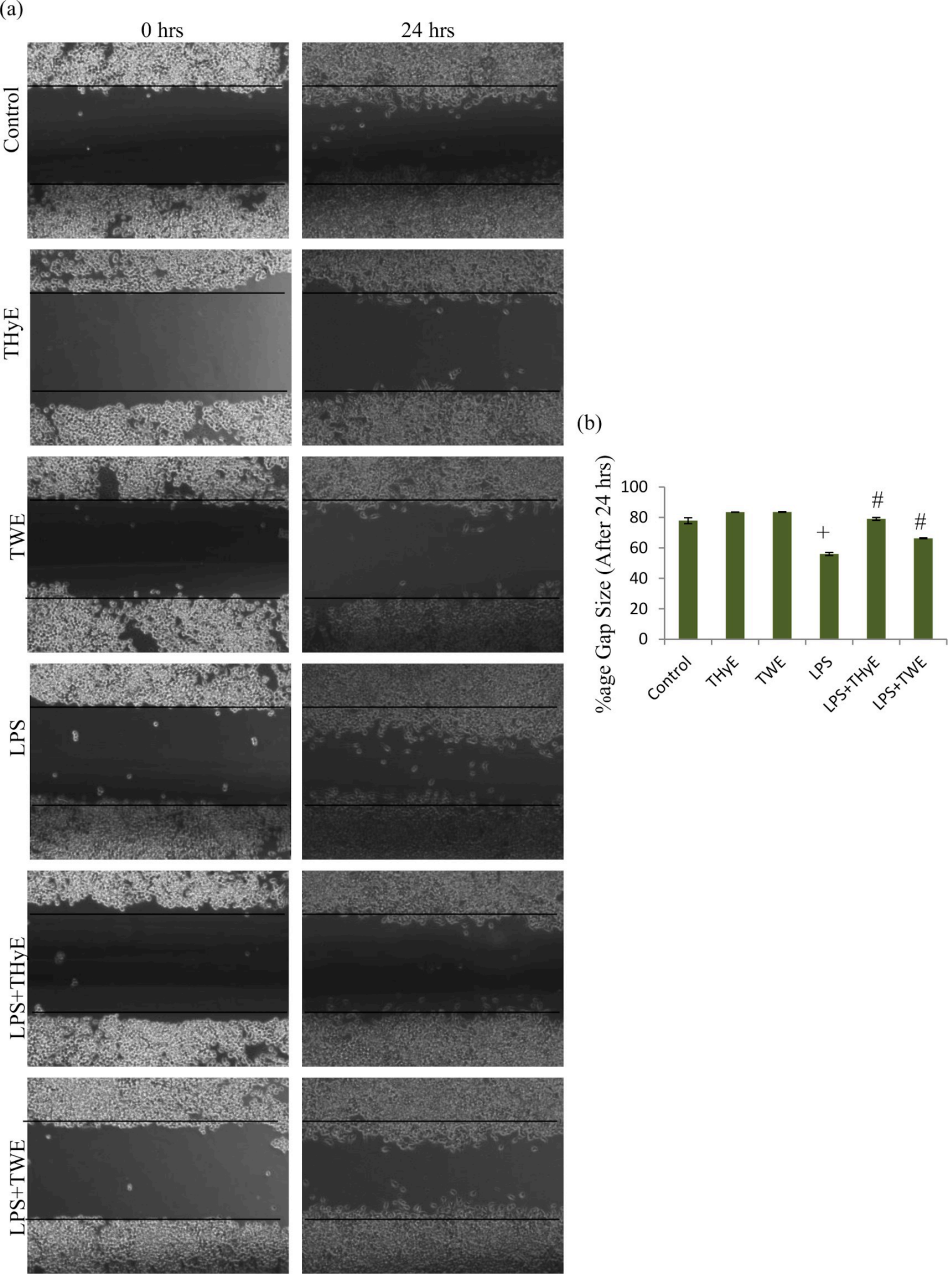
THyE and TWE attenuate the LPS induced migration of the activated BV-2 microglial cells. BV-2 microglial cells were grown as a confluent monolayer, wounded by applying a scratch and treated with THyE (0.2 μg/mL) and TWE (20 μg/mL) with and without activation with LPS. (a) Representative Phase contrast images which were taken at 0 h and 24 h after scratching. (b) Histogram depicting percentage gap size after the 24 h (0 h gap was taken as 100%). ^+^p ≤ 0.001 represents statistically significant difference between control and LPS treated group. ^#^p ≤ 0.001 represents statistically significant difference between LPS and LPS+THyE, LPS+TWE treated groups. *p ≤ 0.001 represents statistically significant difference between control and LPS+THyE, LPS+TWE treated groups.

Rate of microglial migration increases rapidly towards the injury site during inflammatory reactions and is a pathological hallmark responsible for microglial activation. Therefore, to determine the effect of THyE and TWE to inhibit the microglial migration, wound scratch assay was performed and rate of migration into the cell-free scratch area was assessed. Representative phase contrast images taken at 0h and 24h after scratch clearly revealed that ThyE and TWE pretreatment to the LPS activated BV-2 microglial cells reduced their migration into the scratched area (Fig 2A). Histogram depicting gap size clearly shows that LPS treatment reduced gap size up to 55% of the original gap after 24h (taking gap size at 0h as 100%) (Fig 2B). LPS induced reduction in gap size was significantly inhibited up to 79% and 66% of the original gap size after 24h in THyE and TWE pretreated LPS-activated BV-2 microglia respectively, indicating anti-migratory potential of *T*. *indica* extracts (Fig 2B).

### *T*. *indica* extracts and microglial activation

**Fig 3:**
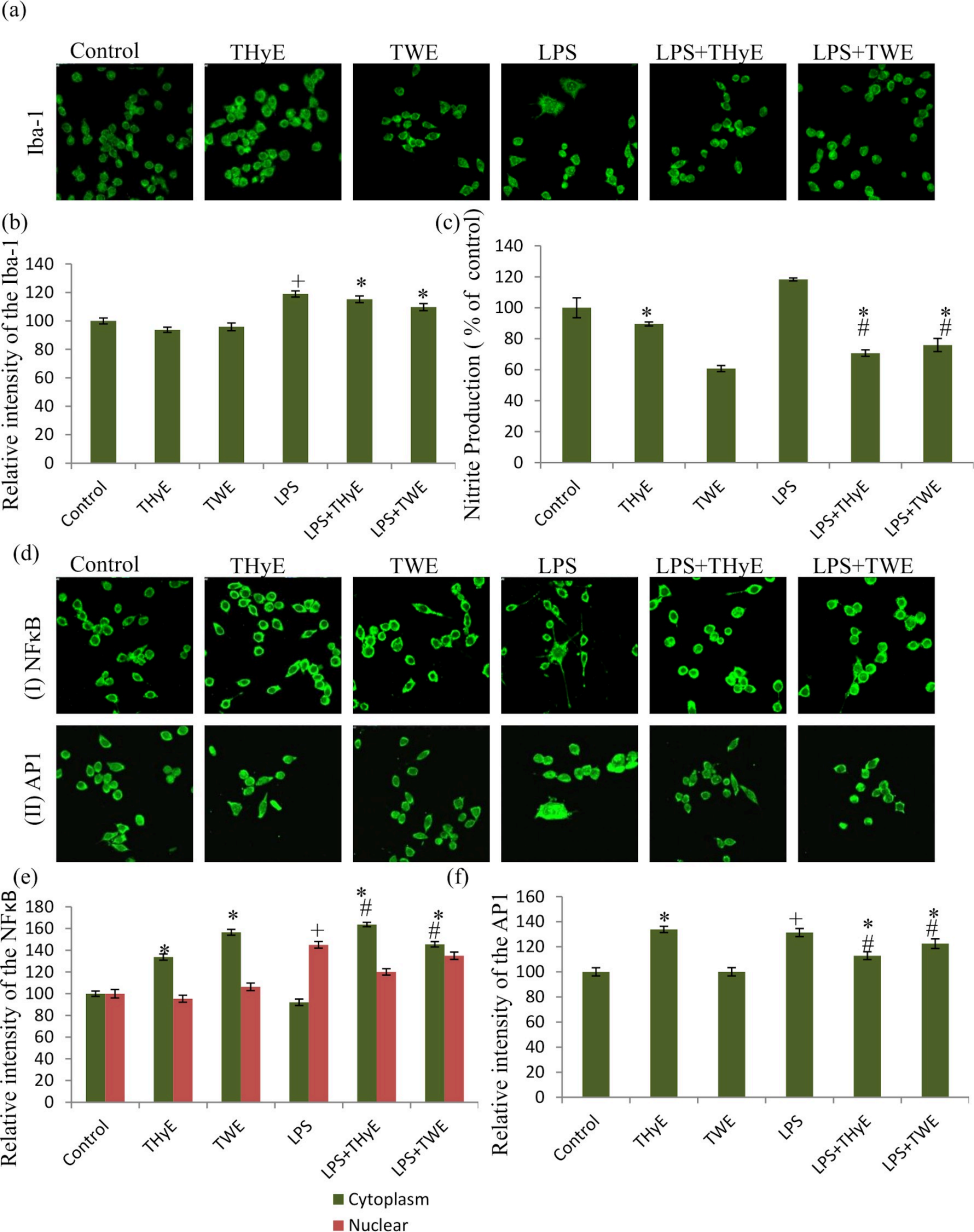
THyE and TWE inhibited microglial activation, nitrite production and expression of inflammatory proteins. (a) Confocal images of Iba-1 immunostaining of the BV-2 microglial cells pretreated with THyE (0.2 μg/mL) and TWE (20 μg/mL) with or without activation with LPS. (b) Histogram depicting the relative optical intensity of Iba-1 in BV-2 microglial cells among the different treated groups. (c) Histogram presenting the relative production of the nitrite in microglial conditioned media among different treated groups as estimated by the Griess Reagent. (d) Representative confocal images of NFκB and AP1 immunostaining of the BV-2 microglial cells pretreated with THyE (0.2 μg/mL) and TWE (20 μg/mL) with or without activation with LPS. (e) Histogram depicting the relative cytoplasmic and nuclear expression of NFκB in BV-2 microglial cells among the different treated groups. (f) Histogram depicting the relative optical intensity of AP1 in BV-2 microglial cells among the different treated groups. Images were captured using A1R Nikon Confocal laser scanning microscope at 60X objective magnification (Scale bar 50μm). ^+^p ≤ 0.001 represents statistically significant difference between control and LPS treated group. ^#^p ≤ 0.001 represents statistically significant difference between LPS and LPS+THyE, LPS+TWE treated groups. *p ≤ 0.001 represents statistically significant difference between control and LPS+THyE, LPS+TWE treated groups.

Microglial activation is generally associated with up-regulated expression of nitrooxidative enzymes like inducible nitric oxide synthetase (iNOS) which lead to production of nitric oxide responsible for the release of various reactive nitrogen species and their derivatives. Thus, LPS induced production of nitrite in BV-2 microglial cells was assayed by Griess reagent which works on azo coupling reaction between the diazonium species produced from naphthylethylenediamine and sulfanilamide in presence of NO_2_. LPS treatment significantly elevated the release of nitrite up to 118% as compared with control group (taken as 100%). While, THyE and TWE pretreatment attenuated the production of nitrite significantly up to 71% and 76%, respectively (Fig 3C).

At molecular level, LPS induced microglial activation was ascertained by the expression of Iba-1, an ionized calcium-binding adaptor protein which helped to distinguish between normal and activated microglial cells [34]. THyE and TWE pretreatment suppressed the LPS induced increase in expression of Iba-1 in BV-2 microglial cells (Fig 3A). Data in histograms depict the relative intensity of Iba-1 expression in BV-2 cells and suggests that LPS treatment significantly up-regulated its expression up to 119% as compared with control group (taken as 100%) (Fig 3B). LPS induced increase in Iba-1 expression was marginally reduced up to 104% and 110% in THyE and TWE pretreated microglial cells, respectively.

Further to elucidate the molecular mechanism underlying the anti-inflammatory potential of *T*. *indica* extracts, we studied the expression of various proteins involved in inflammatory signaling pathways. NFκB and AP1 are the key molecules of LPS induced NFκB and MAPK pathways whose altered expression plays important role in determining the inflammatory state of the cell. LPS treatment significantly up-regulated the nuclear expression of NFκB as compared with control group which was found to be inhibited in the THyE and TWE pretreated LPS activated BV-2 microglia. Similarly, ThyE and TWE pretreatment significantly down-regulated the LPS induced expression of AP1 in BV-2 microglial cells as shown in the confocal images (Fig 3D, row a and b) and their intensity plots (Fig 3E and 3F).

### Effect of cytokinins on multiple shoot proliferation

**Fig 4:**
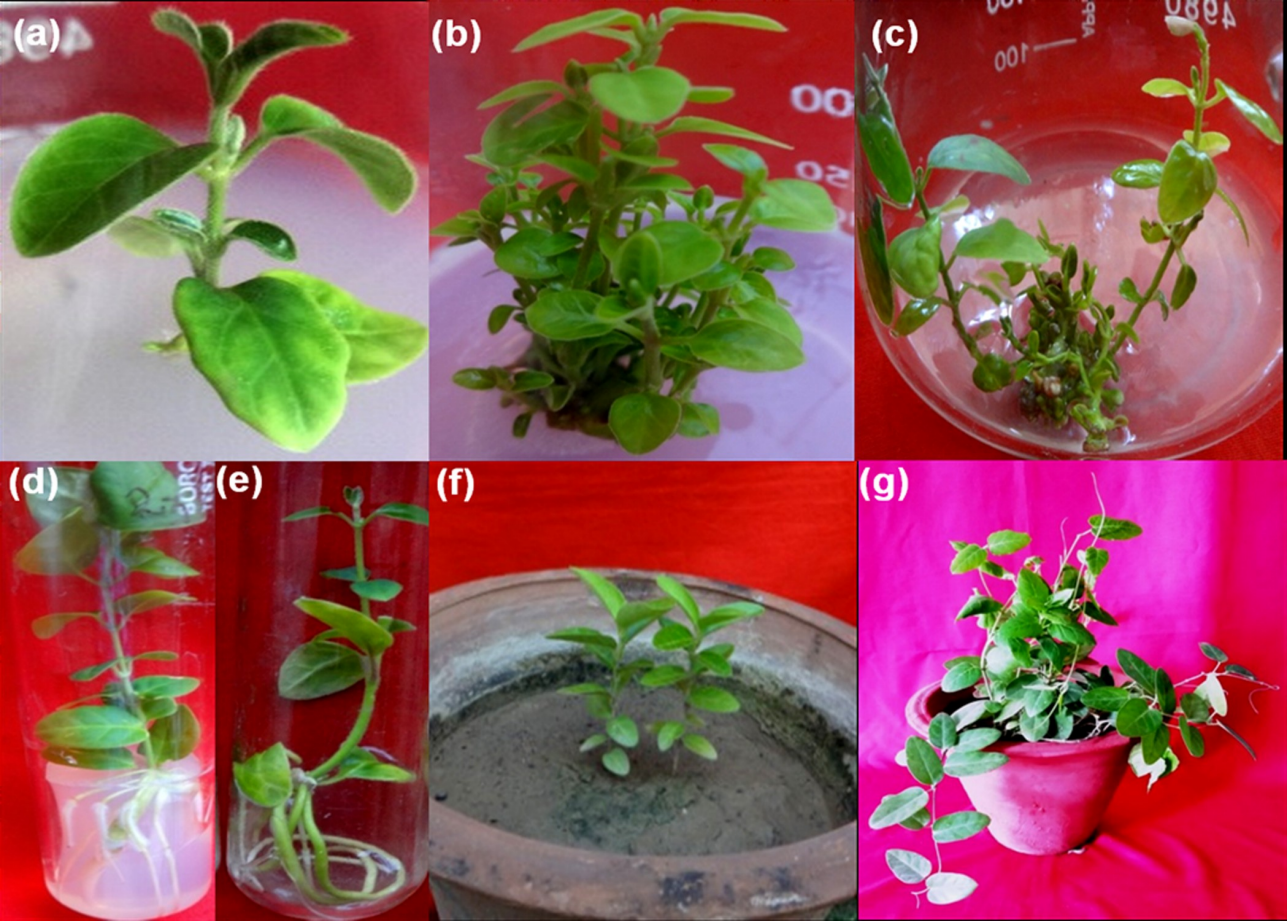
*In vitro* micropropagation of *Tylophora indica*. (a) Initial bud break from the nodal segment on solid MS+ BAP (15μM) after 10 days of culturing. (b) Numerous shoots formed on the same medium after 4 weeks. (c) Axillary shoot proliferation in liquid MS + BAP (10 μM). (d) Rooting of regenerated shoots on solid MS + IBA (10μM). (e) A bunch of roots formed in liquid MS + IBA (5μM). (f) Hardened plantlets in green house. (g) A 4-months-old well acclimatized plant in the field conditions.

The effects of two major cytokinins BAP and KN was assessed on multiple shoot proliferation from nodal explants on both solid and liquid MS media. In solid MS medium, 15 μM treatment of BAP was observed to show vigorous growth of shoots as the number of shoots (13.00 ± 0.25) and shoot biomass (1940 ± 0.06mg) was found to be significantly higher as compared with other treatments (Table 2). However, in liquid MS medium, 10μM of BAP was found to be best in terms of shoot numbers (12.75 ± 0.47) and shoot biomass (2543 ± 0.168mg) as depicted in Table 3. The initial bud break occurred after 10–12 days of inoculation (Fig 4A) leading to the formation of 13–14 shoots from the axillary position on both solid and liquid media after 4 weeks (Fig 4B and 4C) in nearly 80% cultures. The shoots grew further forming well developed leaves.

**Table 2:** Effect of benzyl amino purine (BAP) and kinetin (KN) on multiple shoot proliferation on agar gelled MS medium.

Concentration(μM)	Mean number of shoots per explant	Shoot biomass (mg)
Control	1.25 ± 0.25^*i*^	.180.0 ± 0.02^*i*^
BAP (1)	2.50 ± 0.28^*g*^	490.0 ± 0.03^*g*^
BAP (2.5)	3.75 ± 0.25^*f*^	810.0 ± 0.12^*e*^
BAP (5)	4.75 ± 0.62^*f*^	1560.0 ± 0.05^*b*^
BAP (10)	7.25 ± 0.70^*c*^	1750.0 ± 0.02^*a*^
BAP (15)	13.00 ± 0.25^*a*^	1940.0 ± 0.06^*a*^
KN (1)	1.50 ± 0.28^*h*^	400.0 ± 0.01^*h*^
KN (2.5)	3.50 ± 0.50^*f*^	530.0 ± 0.02^*f*^
KN (5)	5.25 ± 0.25^*e*^	710.0 ± 0.03^*d*^
KN (10)	6.50 ± 0.28^*d*^	1110.0 ± 0.11^*c*^
KN (15)	8.50 ± 0.50^*b*^	1810.0 ± 0.03^*a*^

Data recorded after 4 week of subculture; values represent mean ± SE of five replicates of each concentration. Different letters within the columns are significantly different from each other (Tukey, P<0.05).

**Table 3:** Effect of benzyl amino purine (BAP) on multiple shoot proliferation in liquid MS medium.

Concentration(μM)	Mean number of shoots per explants	Shoot biomass (mg)
Control	0.55 ± 0.26^*f*^	300.0 ± 0.13^*e*^
BAP(2.5)	3.25 ± 0.25^*e*^	963.0 ± 0.02^*c*^
BAP(5)	7.50 ± 0.28^*b*^	2090.0 ± 0.04^*b*^
BAP(10)	12.75 ± 0.47^*a*^	2543.0 ± 0.16^*a*^
BAP(15)	6.00 ± 0.70^*c*^	693.0 ± 0.01^*d*^
BAP(20)	4.00 ± 0.40^*d*^	678.0 ± 0.02^*d*^

Data recorded after 4 week of subculture; values represent mean ± SE of five replicates of each concentration. Different letters within the columns are significantly different from each other (Tukey, P<0.05).

### Rooting of regenerated shoots and their acclimatization

For root induction, the regenerated shoots (4-5cm) were transferred to different root inducing media comprising of different concentrations of IAA, NAA or IBA. Among these, the best rooting response on solid MS medium occurred on 15μM IBA where rooting initiated after 12–15 days of culturing resulting in the formation of 5.50 ± 0.922 roots per shoot as depicted in Fig 4D and Table 4. However, on liquid MS medium, maximum number of roots 5.33 ± 1.202 (Table 5) were formed on lower concentration of 5μM IBA (Fig 4E). The rooted plantlets were successfully acclimatized through successive hardening stages and successfully transferred to natural environmental conditions with 90% survival rate with no phenotypic variations observed. Initially, the rooted plantlets were acclimatized on sterile sand and soil mixture and vermicompost (1:1) under high humidity in the growth room. The plants were carefully monitored and after 5 weeks they were transferred to green house bench (Fig 4F) for another 2 weeks before their final transfer to open field conditions. Fig 4G depicts a 4- month- old well acclimatized plant in the soil.

**Table 4:** Rooting of microshoots on solid MS medium supplemented with different auxins.

Concentration of PGRs (μM)	No. of roots formed per shoot
MS0	2.14 ± 0.34^*b*^
IBA(1)	2.66 ± 0.49^*b*^
IBA(2.5)	4.16 ± 0.65^*a*^
IBA(5)	5.16 ± 0.70^*a*^
IBA(10)	4.50 ± 0.95^*a*^
IBA(15)	5.50 ± 0.92^*a*^
IAA(1)	2.16 ± 0.30^*b*^
IAA(2.5)	2.00 ± 0.36^*b*^
IAA(5)	3.66 ± 0.33^*b*^
IAA(10)	3.66 ± 0.55^*b*^
IAA(15)	3.83 ± 0.47^*b*^
NAA(1)	3.16 ± 0.65^*b*^
NAA(2.5)	4.16 ± 0.91^*a*^
NAA(5)	2.50 ± 0.42^*b*^
NAA(10)	2.00 ± 0.25^*b*^
NAA(15)	1.66 ± 0.33^*b*^

Data recorded after 4 week of subculture; values represent mean ± SE of five replicates of each concentration. Different letters within the columns are significantly different from each other (Tukey, P<0.05). (IBA: Indole-3-butyric acid; IAA: Indole-3-acetic acid; NAA: 1-Naphthaleneacetic acid).

**Table 5:** Rooting of microshoots in liquid MS medium supplemented with different auxins.

Concentration of PGRs(μM)	No. of roots formed per shoot
MS0	3.00 ± 0.57^*d*^
IBA(1)	1.66 ± 0.33^*f*^
IBA(2.5)	2.33 ± 0.33^*e*^
IBA(5)	5.33 ± 1.20^*a*^
IBA(10)	3.33 ± 0.33^*c*^
IBA(15)	4.66 ± 0.66^*b*^
IAA(1)	1.66 ± 0.33^*f*^
IAA(2.5)	2.83 ± 0.30^*e*^
IAA(5)	2.66 ± 0.33^*e*^
IAA(10)	2.66 ± 0.33^*e*^
IAA(15)	3.00 ± 0.57^*d*^

Data recorded after 4 week of subculture; values represent mean ± SE of five replicates of each concentration. Different letters within the columns are significantly different from each other (Tukey, P<0.05). (IBA: Indole-3-butyric acid; IAA: Indole-3-acetic acid).

### RAPD analysis

**Fig 5:**
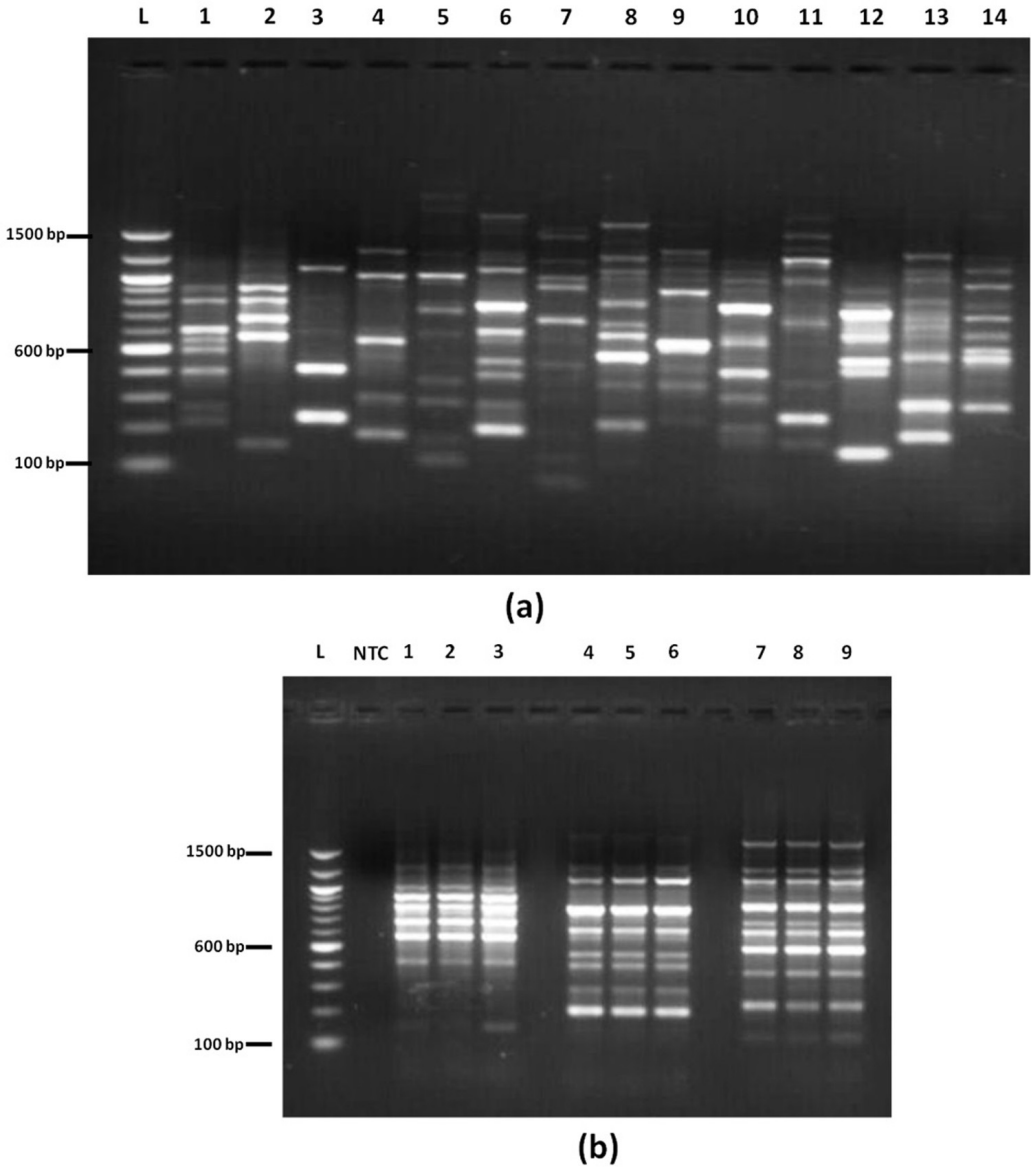
RAPD analysis of micropropagated *Tylophora indica* plants. (a) RAPD pattern as revealed by 14 different RAPD primers screened for number of scorable bands. L: 100 bp ladder; 1: OPA-05; 2: OPA-06; 3: OPA-08; 4: OPA-14; 5: OPB-03; 6: OPB-10; 7: OPB-13; 8: OPC-08; 9: OPC-10; 10: OPC-15; 11: OPD-12; 12: OPD-20; 13: OPG-03; 14: OPH-02. (b) RAPD pattern of mother, *in vitro* and hardened plant of *T*. *indica*. (L: 100 bp ladder; NTC: no template control; 1–3: mother, *in vitro* and hardened plant, respectively with primer OPA-06; 5–7: mother, *in vitro* and hardened plant, respectively with primer OPB-10; 9–11: mother, *in vitro* and hardened plant respectively, with primer OPC-08.

To check the genetic integrity of *in vitro* raised plants, RAPD patterns were compared with that of mother plant using 3 RAPD primers (OPA-06, OPB-10 and OPC-08) screened on the basis of number of scorable bands they produced. *In vitro* formed plants showed uniformity in RAPD profiles under same conditions indicating the genetic stability during the different stages of micropropagation. Each primer generated varied number of bands with the amplification ranging between 0.3kb and 1.5kb (Fig 5). Reproducibility of RAPD pattern was very consistent as identical RAPD patterns were obtained. DNA bands that could not be readily distinguished as present or absent because of their low visual intensity were considered ambiguous markers and were not scored (Table 1).

## Discussion

Accumulating evidence suggests that suppression of microglia mediated neuroinflammation is considered as an intriguing therapeutic target to halt or prevent the development of neurodegenerative diseases [12, 14, 15]. Therefore, the current study was aimed to elucidate anti-neuroinflammatory potential of *T*. *indica* leaf extracts against LPS induced neuroinflammation using BV-2 microglial cells as the *in vitro* model system. MTT assay revealed that treatment of BV-2 cells with both water (TWE) and hydroalcoholic (THyE) leaf extracts for 48h limit their proliferation rate. Since THyE and TWE treatment decreases the cell viability beyond the concentration ≥ 0.5μg/mL and ≥ 30μg/mL, respectively, in a dose dependent manner, so 0.2μg/mL and 20μg/mL dose respectively for THyE and TWE were used for further experimentation. 100ng/mL concentration of LPS was used as an effective activation stimulus for the BV-2 microglial cells as reported previously [32].

In the resting state, microglial cells function as Sentinels, nurturers and warriors, thus maintaining the CNS homeostasis. In response to any insult, microglial cells undergo phenotypic, biochemical and molecular alterations to acquire the activated state [35, 36]. LPS treated BV-2 microglial cells showed the phenotypic switch from ramified to amoeboid morphology with filopodia like cytoplasmic processes as revealed from α-tubulin immunostaining (Fig 1C). THyE and TWE pretreatment were observed to prevent these alterations and cells showed normal surveilling morphology. At the molecular level, THyE and TWE pretreatment down-regulated the expression of Iba-1, an indicator of microglial activation which was up-regulated in LPS treated BV-2 microglial cells. Activated microglial cells showed enhanced migration to reach the site of injury [37]. Wound scratch assay showed increased microglial cells migration rate in LPS challenged cultures, as indicated by gap size reduction from 0 to 24h of treatment (Fig 2). THyE and TWE pretreatment was seen to slow down the migration of BV-2 microglial cells toward the lesion site. Activated microglial cells secrete various inflammatory or neurotoxic products like RNS, ROS, pro-inflammatory cytokines ‘etc’, which act in autocrine or paracrine fashion or as secondary messengers to amplify the production of these factors and increase the degree of microglial activation leading to neurodegeneration and cognitive deficits in neurodegenerative diseases [12, 13]. *T*. *indica* extracts pretreatment also attenuated LPS induced nitrite production in BV-2 microglial cells and this activity may be attributed to the presence of the phenanthroindolizidine alkaloids in these extracts. Two of these alkaloids, tylophorine and ficuseptine A have been earlier reported to suppress the nitric oxide production in the LPS/IFN-γ stimulated RAW 264.7 cells via inhibiting the expression of inducible nitric oxide synthase and activation of its promoter NFκB and AP1 activity [20]. Withaferin A, an active phytochemical of Ashwagandha has also been reported to inhibit the iNOS expression and NO production in Raw 264.7 macrophages [38]. Similarly, *Toona sinensis* also targeted production of nitric oxide as an underlying anti-inflammatory mechanism against microglial mediated neuroinflammation [39].

**Fig 6:**
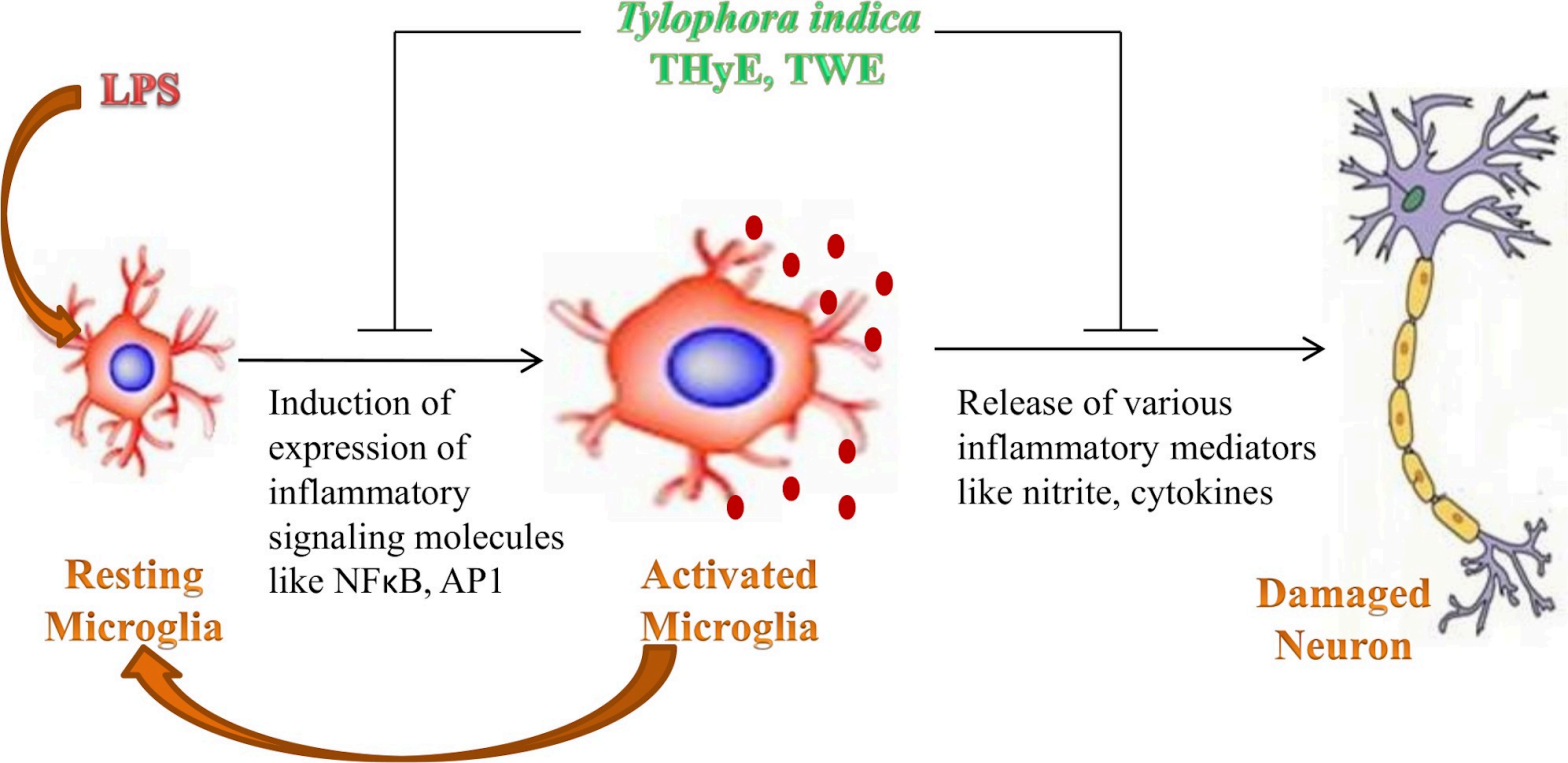
Graphical representation depicting the anti-neuroinflammatory activity of *Tylophora indica* against LPS induced inflammatory responses and possible molecular mechanisms.

Activation of NFκB and MAPK pathways are known to be implicated in LPS induced inflammatory responses [40]. In the present study, *T*. *indica* extracts pretreatment was observed to inhibit the LPS induced increase in expression and nuclear translocation of transcription factor NFκB (Fig 6). NFκB activation is critical for the initiation and progression of the inflammatory responses via transcription of various inflammatory genes including cytokines, chemokines, nitro-oxidative products ‘etc’ [41]. THyE and TWE pretreatment also suppressed the LPS induced transcriptional activation of AP1 in BV-2 microglial cells. The co-stimulation of both NFκB and AP1 has synergistic effect on the inflammatory reactions [42, 43]. Therefore, it may be suggested that THyE and TWE targeted both AP1 and NFκB as the underlying mechanism responsible for the anti-neuroinflammatory potential of the *T*. *indica*.

Many potent anti-inflammatory herbs and their products like Ashwagandha, curcumin, resveratrol have been reported to prevent LPS induced microglial proliferation, migration and production of inflammatory mediators via inhibiting both NFκB and AP1 signaling cascades [32, 44, 45, 46]. *T*. *indica* leaf extract has also been reported to have anti-inflammatory effect on carrageenin induced hind paw odema and cotton pellet granuloma in albino rats [47]. Moreover, anti-inflammatory effect of plant extract was observed to be more effective than commercially available non steroidal anti-inflammatory drug Indomethacin in both acute and subacute model of inflammation. Collectively, these observations suggests that *T*. *indica* leaf extracts could effectively suppress the LPS induced microglial activation, migration and subsequent neuroinflammatory responses (Fig 6). This anti-neuroinflammatory potential of *T*. *indica* could be attributed to the presence of therapeutically important alkaloids such as tylophorine, tylophorinine and tylophorinidine in the THyE and TWE leaf extracts of this plant [2, 4]. Thus, this plant may prove to be a safe alternative to the conventional NSAIDs used for the treatment of the neurodegenerative diseases.

Lack of cultivation practices and indiscriminate harvesting of its roots for preparation of drug has threatened the very existence of *T*. *indica*. Thus, to ensure the ample supply of this herb without overexploiting its natural habitats, it is critical to develop an efficient micropropagation system for its large scale multiplication. In the present work, a cost effective method for the *in vitro* propagation for *T*. *indica* has been developed that will fulfill its increasing demand in future and will also help in its conservation. The cytokinins are well known to play a critical role in the regulation of shoot multiplication in plants [48]. In the present investigation, out of two cytokinins viz BAP and KN tested, 15μM of BAP was found to be most efficient for multiple shoot proliferation on solid MS medium as number of shoots and shoot biomass was observed to be highest at this concentration. The effectiveness of cytokinins especially BAP in promoting axillary shoot proliferation in many medicinal plants is well documented in conformity with our results. Earlier, the highest number (8.6 ± 0.71) of shoots from nodal segments in *T*. *indica* were recorded on MS medium supplemented with 2.5 μM IBA, 0.5 μM NAA and 100mg/l ascorbic acid [25]. In contrast, we have established a more efficient protocol forming 12–13 shoots per nodal explants on both solid and liquid media. However, reduced rate of shoot proliferation at 15μM of KN may be due to its weak potential to induce shoot multiplication [49, 50].

One of the major challenges involved in the scale-up of *in vitro* micropropagation method is the cost involved in the process. To address this challenge, development of liquid micropropagation method is considered to be critical as it significantly reduces the cost of the process as well as ensures efficient direct uptake of nutrients which leads to better growth [51]. Thus, in the present work, shoot proliferation has also been standardized in liquid culture system using 20ml liquid MS medium supplemented with 10μM BAP which was found to be most effective for shoot multiplication. At higher concentrations of BAP (15 and 20μM), vitrification of shoots was invariably observed in liquid medium. This observation aligns with the previous reports where higher concentrations of BAP have been shown to cause vitrification in many medicinal plants [52]. However, to the best of our knowledge, this is the first report regarding *T*. *indica* where a liquid medium has been used for its *in vitro* propagation. Besides being cost effective, it was more efficient system as the number of shoots produced per nodal explant was more as compared with previous reports.

Auxins are well known as rooting hormones and are routinely used for initiating root development of *in vitro* shoots [50, 53]. It positively regulates the various carbohydrate, nitrogen, protein and polyphenolic metabolic pathways which are well known to influence an array of pathways involved in root growth and development [54]. Out of all the auxins tested, IBA proved to be the best for the induction of roots both on solid and liquid media where excellent rooting response was observed in 100% of cultures. Significant root induction also occurred on IAA and NAA supplemented MS medium. Both the observations are well supported by references from the literature. Thomas and Philip and Faisal and Anis have reported IBA to be optimal for rooting in the regenerated shoots of *Tylophora indica* [28, 55] whereas Bera and Roy recorded IAA to be the best for root induction [56]. On the other hand, Kaur et al. reported the highest frequency (90–95%) of rooting on auxin free half strength MS medium alone [29,30,31]. In the present study, a successful attempt has been made to acclimatize the tissue culture raised plants of *T*. *indica* using sand and soil mixture and vermicompost in the ratio of 1:1 and were successfully transferred to natural environmental conditions with 90% survivability. The present results are in accordance with the earlier observations on *Tylophora* where soil: vermicompost treatment was found to be most cost effective and was almost in par with the biofertilizers supplemented potting mixture in terms of survival percentage of plants [57].

One of the undesirable consequences of *in vitro* propagation is the occurrence of somaclonal variations which limits the utility of micropropagation system [58]. As in the present study, *T*. *indica* shoots were subcultured multiple times, therefore it is critical to ensure the genetic stability of micropropagated plantlets for the successful transfer of the present protocol to the commercial level. Genetic stability of micropropagated plants is usually appraised using DNA-based molecular markers such as amplified fragment length polymorphism (AFLP), random amplification of polymorphic DNA (RAPD), restriction fragment length polymorphism (RFLP), inter simple sequence repeat (ISSR), simple sequence repeat (SSR), sequence related amplified polymorphism (SRAP) [59, 60]. In the present work, clonal fidelity of *in vitro* propagated shoots was assessed using RAPD approach being cost effective and easier to perform [61, 62]. Upon analysis, mother plant, *in vitro* plants and hardened plants showed uniformity in RAPD profiles under same amplification conditions indicating the genetic identity during the different stages of micropropagation.
